# Current perspectives on mechanisms of ribonucleotide incorporation and processing in mammalian DNA

**DOI:** 10.1186/s41021-019-0118-7

**Published:** 2019-01-25

**Authors:** Akira Sassa, Manabu Yasui, Masamitsu Honma

**Affiliations:** 1 Department of Biology, Graduate School of Science, Chiba University, Chiba, 263-8522 Japan; 2 Division of Genetics and Mutagenesis, National Institute of Health Sciences, 3-25-26 Tonomachi, Kawasaki-ku, Kawasaki 210-9501 Japan

**Keywords:** Ribonucleotide, Genome instability, DNA polymerase, DNA repair

## Abstract

Ribonucleotides, which are RNA precursors, are often incorporated into DNA during replication. Although embedded ribonucleotides in the genome are efficiently removed by canonical ribonucleotide excision repair (RER), inactivation of RER causes genomic ribonucleotide accumulation, leading to various abnormalities in cells. Mutation of genes encoding factors involved in RER is associated with the neuroinflammatory autoimmune disorder Aicardi–Goutières syndrome. Over the last decade, the biological impact of ribonucleotides in the genome has attracted much attention. In the present review, we particularly focus on recent studies that have elucidated possible mechanisms of ribonucleotide incorporation and repair and their significance in mammals.

## Background

In eukaryotic cells, the concentrations of ribonucleotide triphosphates (rNTPs), i.e., RNA precursors, are approximately two orders of magnitude higher than those of DNA precursors, deoxyribonucleotide triphosphates (dNTPs) [1, 2]. Although DNA polymerases (pols) can accurately discriminate the correct substrate dNTPs against rNTPs, the great abundance of rNTPs in cellular nucleotide pools enables them to be incorporated into genomic DNA. Indeed, numerous rNTPs are incorporated into the genome; approximately 13,000 and > 1000,000 ribonucleotides are embedded into the genomes of yeast and mouse embryonic fibroblast cells, respectively [3, 4]. In humans, hypomorphic mutations of the genes encoding subunits of RNase H2, the enzyme essential for initiation of canonical ribonucleotide excision repair (RER), are associated with the serious autoimmune disease Aicardi–Goutières syndrome (AGS) [5]. The AGS autoimmune phenotype is believed to be caused by the accumulation of endogenous nucleic acid species, which activate intracellular Toll-like receptors, and/or DNA damage responses induced by the embedded ribonucleotides, stimulating interferon production in RNase H2-compromised cells [6]. In mouse models, early embryonic lethality results from the complete disruption of RNase H2 [3, 7]. Additionally, tissue-specific inactivation of RNase H2 can progress to tumorigenesis [8, 9]. Mammalian cells deficient in RER accumulate ribonucleotides in the genome and display various abnormalities, such as DNA replication delay, enhanced DNA damage, chronic activation of DNA damage responses, and epigenetic dysfunction [3, 7, 10–12]. Thus, genomic ribonucleotide accumulation is a disastrous event in cells, and molecular mechanisms underlying ribonucleotide-induced genome instability have been of a great interest over the last decade. Essential studies in this field have been well summarized in several reviews [13–19]. In this article, we focused on mammals in particular and recent research that has investigated the possible mechanisms underlying ribonucleotide incorporation and their processing pathways has been described.

## Review

### Source of ribonucleotide incorporation into DNA

Eukaryotic DNA pols are classified into six families (A, B, X, Y, RT, and AEP) on the basis of amino acid sequence comparisons [20, 21]; family A (pols γ, θ, and ν), family B (pols α, δ, ε, and ζ), family X (pols β, λ, μ, and TdT), family Y (pols η, κ, ι, and Rev1), family RT including telomerase, and family AEP including PrimPol. Most pols possess a conserved “steric gate” amino acid residue, which prevents ribonucleotide incorporation into DNA [22]. Although pols β and λ lack an aromatic steric gate amino acid side chain, both pols utilize a protein backbone segment to discriminate among sugars [23–25].

Although pols have a discrimination system against rNTPs, they can incorporate rNTPs into DNA at a non-negligible rate. For the human replicative pol α from family B, rNTPs are inserted with a 500-fold lower frequency than dNTPs during DNA synthesis [26]. The other replicative pols, δ and ε, are prone to incorporate rNTPs at physiological nucleotide concentrations similar to those of yeast replicative pols that incorporate one ribonucleotide for every thousands of deoxyribonucleotides [27, 28]. Therefore, millions of ribonucleotides may be embedded into the human genome. Notably, 3′-exonuclease activities of these pols cannot efficiently remove the inserted ribonucleotides [27, 28], which suggests that the proofreading during replication does not protect the genome from the aberrant ribonucleotide incorporation.

The mitochondrial pol γ, a member of family A, discriminates rNTPs with 1000- to 77,000-fold preference for dNTPs depending on the identity of nucleotides [26, 29]. As observed in family B pols, the 3′-exonuclease activity of pol γ does not contribute to the protection from ribonucleotide incorporation [30]. Based on previous studies, for 16.5 kb of mitochondrial DNA (mtDNA), pol γ is predicted to incorporate roughly 10–20 ribonucleotides during replication. However, the number of ribonucleotides in mtDNA (54, 36, and 65 ribonucleotides in one mtDNA molecule of human fibroblasts, HeLa cells, and mouse liver, respectively) was shown to be much higher than the expected frequency [30, 31]. This difference is expected to result from the presence of the other pols participating in mtDNA replication and/or the influence of varying nucleotide concentrations inside mitochondria [30].

Family X pols, involved in DNA repair processes such as base excision repair (BER) and non-homologous end joining (NHEJ), have also been suggested to play roles in inserting ribonucleotides into DNA. Pols β and λ have substrate selectivity in the range of 3,000- to 50,000-fold preference for dNTPs in comparison with rNTPs [22]. Although they strongly discriminate against ribonucleotides, a recent study showed that pol β, rather than pol λ, has an impact on the activity of ribonucleotide insertion opposite 7,8-dihydro-8-oxo-2′-deoxyguanosine (8-oxo-dG), a base resulting from oxidative damage, in cellular extracts [32]. Additionally, oxidative ribonucleotide 8-oxo-rGTP can be utilized as a substrate for DNA synthesis by pol β [33]. Notably, pol μ and TdT, unlike other pols, favorably incorporate rNTPs into DNA (only 1- to 10-fold discrimination against rNTPs) [22, 34]. Importantly, ribonucleotides are primarily utilized by both pols during NHEJ in cells [35], leading to beneficial consequences for DNA strand break repair; the insertion of ribonucleotides increases the fidelity of pol μ and promotes the ligation step during NHEJ [35, 36]. Although DNA repair processes, as well as DNA replication, can be sources of ribonucleotide incorporation, the transient presence of ribonucleotides contributes to the efficient repair of DNA maintaining genome integrity.

Family Y pols can replicate across DNA lesions via a process known as translesion DNA synthesis (TLS). Despite the presence of the steric gate residue in the active site [37–39], TLS pols can insert rNTPs into DNA in the following specific situations [38, 40]: Pol ι can incorporate rNTPs opposite undamaged template DNA depending on the sequence context. During TLS, the insertion of rNTPs by Pol ι is also observed across damaged DNA such as an abasic site (AP-site) and 8-oxo-dG. Another TLS Pol η can insert rCTP opposite 8-oxo-dG and cisplatin intrastrand guanine crosslinks. In addition, the activity of RNase H2-mediated cleavage of the inserted ribonucleotide decreases in the presence of these types of DNA damage. Thus, the TLS pathway may contribute to genomic ribonucleotide accumulation.

### Repair/tolerance mechanisms of embedded ribonucleotides

#### RNase H2-initiated ribonucleotide excision repair

**Fig. 1:**
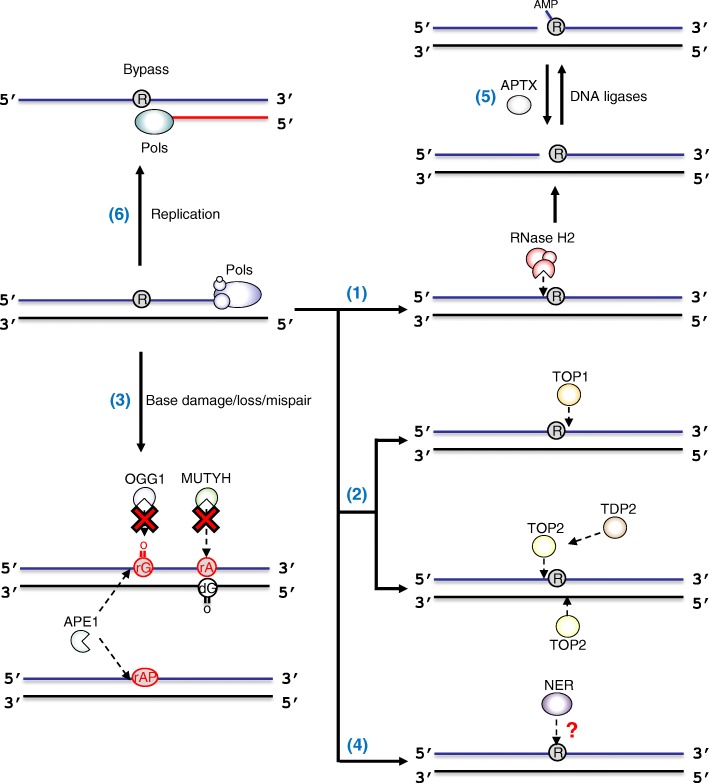
Overview of processing mechanisms of ribonucleotides embedded in DNA. (1) Embedded ribonucleotides are repaired by RNase H2-dependent RER. (2) In the absence of RNase H2, ribonucleotides in DNA are processed by topoisomerases, resulting in genomic instability. (3) The BER factor APE1 excises the damaged ribonucleotides in DNA. (4) The involvement of NER on ribonucleotide removal is under debate. (5) APTX resolves abortive ligation intermediates created at 5′-ribonucleotide termini. (6) Ribonucleotides on the template DNA strand impact on DNA synthesis

Embedded ribonucleotides are primarily repaired by RNase H2-mediated RER (Fig. 1 (1)) [41]. In vitro studies have revealed the detailed mechanism underlying the RER pathway: RNase H2 recognizes the ribonucleotide in DNA and cuts the DNA 5′-phosphodiester bond of the ribonucleotide [42, 43]. This incision reaction is followed by strand displacement synthesis by pols δ or ε, flap DNA cleavage by flap endonuclease FEN1 or the exonuclease Exo1, and nick sealing by DNA ligase I [41].

Eukaryotic RNase H2 is a heteromeric complex containing a catalytic subunit RNASEH2A and auxiliary subunits RNASEH2B and RNASEH2C [43]. RNASEH2B physically interacts with PCNA via the PCNA-interacting motif [44], indicating that RER is coupled with DNA replication. Indeed, mammalian cell studies suggest that RNase H2 is recruited and co-localized to replication and repair foci, not only via the interaction of RNASEH2B and PCNA but also via the catalytic site of RNASEH2A [45, 46]. Notably, RNase H2 is constitutively expressed throughout the cell cycle in HeLa cells [3], implying the possible role of RER in replication-independent repair.

Reportedly, RER is required for efficient mismatch repair (MMR). A single ribonucleotide in close proximity to a mismatch is processed by RNase H2 for generating a nick, which provides a strand discrimination signal for MMR of nascent strand replication errors [47, 48]. Hence, as also observed during NHEJ (see the section above) [35], ribonucleotide insertion is not merely an erroneous event occurring during replication, but it is an important biological process in maintaining genome stability.

#### Topoisomerase-mediated excision repair

In the absence of functional RNase H2, the embedded ribonucleotides are repaired by an alternate pathway involving DNA topoisomerase, the enzyme that relaxes negatively supercoiled DNA by transiently cleaving and re-ligating one or both strands of DNA (Fig. 1 (2)) [49–51]. Yeast and human topoisomerase 1 (TOP1) incise the DNA 3′-side of a ribonucleotide, generating a nick and a covalent protein-DNA cleavage complex (TOP1cc) between the TOP1 tyrosyl moiety and the 3′-phosphate of the ribonucleotide [52, 53]. Upon cleavage, the 2′-hydroxyl of the ribose sugar attacks the phosphotyrosyl linkages, generates a 2′,3′-cyclic phosphate, and releases TOP1 [52, 53].

**Fig. 2:**
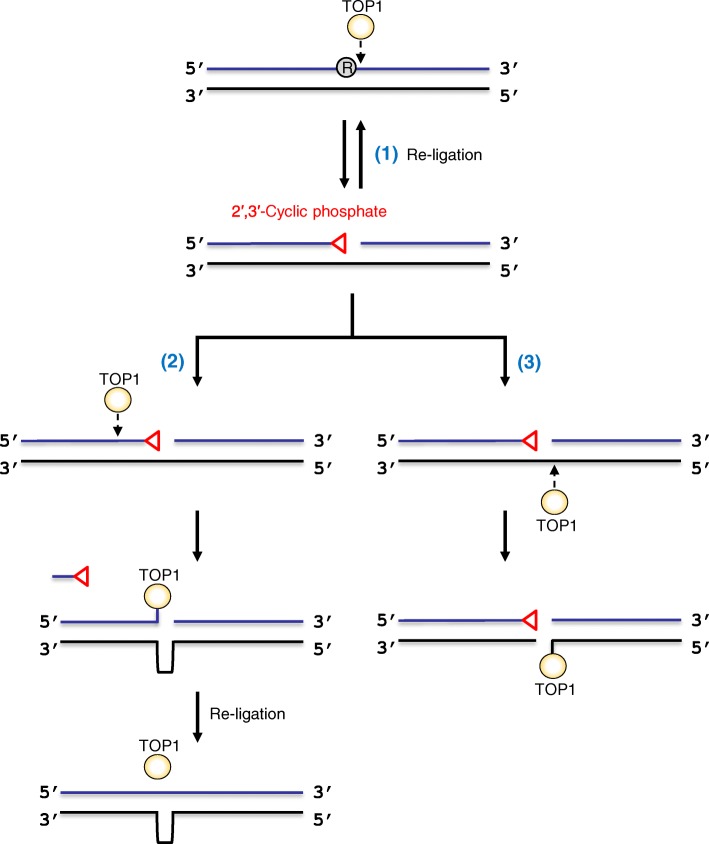
Models depicting the processing of ribonucleotide by mammalian topoisomerase 1. (1) A nick containing 2′,3′-cyclic phosphate and 5′-OH ends is re-ligated by TOP1. (2) Strand cleavage by TOP1 upstream from the nick leads to the formation of a second TOP1cc. Re-ligation across the gap by TOP1 causes a short-deletion. (3) Cleavage of the opposite strand by TOP1 results in the formation of the DNA strand break with TOP1cc at the strand terminus

Recent studies using purified human TOP1 suggest further distinct processing of the released DNA (Fig. 2): (1) re-ligation of the nick; (2) strand cleavage by TOP1 a few nucleotides upstream from the nick, leading to the formation of a second TOP1cc; and (3) sequential cleavage on the opposite strand of the nick [54, 55]. Specifically, the re-ligation of the nick by TOP1 allows a second attempt of the excision repair. Second, TOP1cc formation upstream from the nick leads to the release of a short DNA fragment containing 2′,3′-cyclic phosphate, which generates short deletions at repetitive sequences through TOP1-mediated false ligation. Lastly, cleavage of the opposite strand by TOP1 results in the formation of a severe DNA strand break with TOP1cc at the strand terminus. These models have been supported by studies with yeast TOP1, which induces 2–5-nt deletion mutations at the repetitive sequences, as well as DNA double strand breaks in the genome [54, 56, 57]. Furthermore, mouse and human cells lacking RNase H2 had elevated levels of 53BP1 or phosphorylated histone (γH2AX) foci, indicating the formation of DNA strand breaks in the mammalian genome [3, 7, 10, 12]. According to these studies, a question arises as to whether such deletion mutations can be caused by ribonucleotide accumulation in vivo. Findings of a recent study have revealed that deletions are induced by aberrant ribonucleotide incorporation into mouse mitochondrial DNA [58]. In contrast, base substitutions (T:A → G:C base substitutions at GTG trinucleotides), but not deletion mutations, have been detected through whole exome sequencing of tumor cells derived from *Rnaseh2b* knock-out mice [9]. Taken together, TOP1-dependent ribonucleotide excision repair can be highly mutagenic and possibly induces severe genomic instability in the absence of RER; however, its biological consequences in mammalian cells require further investigation.

On the basis of a recent study, the depletion in TOP1 reduces the number of γH2AX foci in RER-deficient human cells [59], which provides evidence of the false processing of embedded ribonucleotides by TOP1 in mammals. Interestingly, the lack of RNase H2 desensitizes human cells to poly(ADP-ribose) polymerase (PARP) inhibitors that form PARP1-trapping DNA lesions [59]. Therefore, DNA damage created by TOP1-mediated ribonucleotide excision induces PARP1 activation. Because mono-allelic or bi-allelic loss of *RNASEH2B* is frequently observed in chronic lymphocytic leukemia and castration-resistant prostate cancers, genomic ribonucleotides may be a therapeutic target in tumors [59].

It has been reported that the presence of ribonucleotides in DNA stimulates the cleavage activity of type II topoisomerase (TOP2) and leads to the formation of a TOP2 cleavage complex (TOP2cc) at 5′-ribonucleotides [60, 61], possibly causing DNA strand breaks. For repairing this ribonucleotide-induced TOP2cc, TOP2 has to be proteolyzed. The consequent degradation of TOP2cc allows the processing of the TOP2-DNA crosslinks by tyrosyl-DNA phosphodiesterase 2 (TDP2) that hydrolyzes the 5′-tyrosine phosphodiester bonds between DNA 5′-phosphates and the active site tyrosine of TOP2 [61]. Therefore, TDP2 plays a protective role against the toxic effects of ribonucleotide-induced DNA damage in cells.

#### Base excision repair

BER is a primary repair pathway that is involved in correcting damage to endogenous bases such as oxidative and alkylated bases, e.g., 7,8-dihydro-8-oxoguanine and *N*^3^-methyladenine [62, 63]. BER is initiated by excision of the damaged or mismatched base by DNA glycosylases. The AP-site produced is further processed by apurinic/apyrimidinic endonuclease 1 (APE1), which catalyzes the cleavage of the sugar-phosphate backbone 5′ at the AP-site. For the mechanism of BER, the question that arises is whether the embedded ribonucleotides are recognized as the substrate of BER factors (Fig. 1 (3)). Reportedly, 8-oxoguanine DNA glycosylase (OGG1) can bind to an oxidized ribonucleotide, i.e., 8-oxoriboguanosine (8-oxo-rG), in DNA but showed no glycosylase/lyase activity in vitro [64]. Similarly, the human MutY homolog (MUTYH), which removes mispaired adenine opposite 8-oxoguanine, is fully inactive against riboadenosine (rA) paired with 8-oxoguanine [33]. Interestingly, APE1 cleaves an abasic ribonucleotide (rAP-site) in DNA and also has weak endonuclease and 3′-exonuclease activities on the embedded 8-oxo-rG, while mammalian RNase H2 has no activity against either rAP-site or 8-oxo-rG [65]. Therefore, among BER mechanisms, APE1 is a candidate for being the back-up repair mechanism for processing damaged ribonucleotides that cannot be removed by RNase H2.

#### Nucleotide excision repair

Nucleotide excision repair (NER) is involved in the removal of helix-distorting DNA lesions such as UV-induced cyclobutane pyrimidine dimers. Because NER factors can recognize a nearly infinite variety of DNA damages, ribonucleotides misincorporated into DNA may serve as the substrate for NER. The possibility of this alternative repair pathway has been debated among researchers (Fig. 1 (4)) [66]. Purified NER proteins derived from thermophilic eubacteria recognize and excise ribonucleotides in DNA [67]. In *E. coli* cells, the disruption of NER factors increases spontaneous mutagenesis in the absence of RNase HII [67]. However, a recent in vitro study revealed that ribonucleotide-containing DNA is a very poor substrate for purified *E. coli* and human NER systems [68], which indicates that NER is not a major repair pathway in mammals. The precise role of NER in the repair of embedded ribonucleotides is presently being debated.

#### Processing of ribonucleotide-induced abortive ligation

During RER, RNase H2 cleaves the 5′-side of a ribonucleotide and creates a nick, i.e., a RNA-DNA junction. In such conditions, the presence of a ribonucleotide on the 5′-terminus impairs the sealing of the nick by human DNA ligases I and III (Fig. 1 (5)). This abortive ligation results in the formation of a toxic 5′-adenylation (5′-AMP) at the ribonucleotide terminus [69]. Human aprataxin (APTX), the enzyme that removes 5′-AMP from abortive ligation intermediates, has been known to efficiently repair the 5′-AMP at RNA-DNA junctions generated during RER. The study indicated that the potential role of APTX is to protect genome integrity against the complex types of damage that can be generated during RER.

#### DNA synthesis across embedded ribonucleotides

In the absence of RER, the accumulation of ribonucleotides into the genome leads to replication stress in cells [3]. On the basis of in vitro experiments, human replicative pol δ pauses slightly during DNA synthesis across a single ribonucleotide on the template DNA (Fig. 1 (6)) [27]. Although human pol α and mitochondrial pol γ are also able to bypass a template ribonucleotide [30, 64], physiological concentrations of rNTPs have been shown to inhibit DNA synthesis by pol γ [30]. Furthermore, multiple consecutive ribonucleotides hinder the primer extension reaction catalyzed by pol δ [27].

The oxidation of ribonucleotides in DNA can be more problematic for replication; the oxidative ribonucleotide 8-oxo-rG strongly blocks primer extension catalyzed by pol α [64]. For TLS pols, pol κ inefficiently bypasses rG and 8-oxo-rG [64]. Interestingly, pol η rapidly bypasses both undamaged and damaged ribonucleotides [64]. Both TLS pols can bypass 8-oxo-rG in a more error-free manner than 8-oxo-dG. Therefore, the ribonucleotide sugar backbone influences fidelity during TLS. These studies suggest that the ribonucleotides in the genome impede replication by pols, possibly stalling replication forks. In this scenario, TLS pols are required as ribonucleotide-tolerance mechanisms.

## Conclusions

There is increasing interest in the impact of ribonucleotide incorporation into DNA. The possible mechanisms underlying ribonucleotide-induced genomic instability and its consequences to the cell have been reported in numerous in vitro and in vivo studies. The recent noteworthy studies described in this review demonstrated that ribonucleotides that are transiently present in the genome are not only problematic lesions but may also be beneficial to the maintenance of genome integrity. However, the inactivation of canonical RER results in various deleterious effects in cells, which likely result from the unwanted processing of ribonucleotides, and may cause severe symptoms in humans. Further studies will be necessary for providing a better understanding of the biological action of the ribonucleotides, e.g., mutagenic potential, in the mammalian genome.

## Abbreviations

APE1: apurinic/apyrimidinic endonuclease 1
8-oxo-dG: 7,8-dihydro-8-oxo-2'-deoxyguanosine
8-oxo-rG: 8-oxoriboguanosine
AGS: Aicardi–Goutières syndrome
APTX: aprataxin
BER: base excision repair
dNTPs: deoxyribonucleotide triphosphates
MMR: mismatch repair
MUTYH: MutY homologue
NER: Nucleotide excision repair
NHEJ: non-homologous end joining
OGG1: 8-oxoguanine DNA glycosylase
PARP: poly(ADP-ribose) polymerase
pol: DNA polymerase
RER: ribonucleotide excision repair
rNTPs: ribonucleotide triphosphates
TDP2: tyrosyl-DNA phosphodiesterase 2
TOP1: topoisomerase 1
TOP2: type II topoisomerase
